# Highly Mimetic Ex Vivo Lung‐Cancer Spheroid‐Based Physiological Model for Clinical Precision Therapeutics

**DOI:** 10.1002/advs.202206603

**Published:** 2023-04-21

**Authors:** Ming‐You Shie, Hsin‐Yuan Fang, Kai‐Wen Kan, Chia‐Che Ho, Chih‐Yen Tu, Pei‐Chih Lee, Po‐Ren Hsueh, Chia‐Hung Chen, Alvin Kai‐Xing Lee, Ni Tien, Jian‐Xun Chen, Yu‐Cheng Shen, Jan‐Gowth Chang, Yu‐Fang Shen, Ting‐Ju Lin, Ben Wang, Mien‐Chie Hung, Der‐Yang Cho, Yi‐Wen Chen

**Affiliations:** ^1^ School of Dentistry China Medical University Taichung 406040 Taiwan; ^2^ x‐Dimension Center for Medical Research and Translation China Medical University Hospital Taichung 404332 Taiwan; ^3^ Department of Bioinformatics and Medical Engineering Asia University Taichung 41354 Taiwan; ^4^ Department of Thoracic Surgery China Medical University Hospital Taichung City 40447 Taiwan; ^5^ School of Medicine China Medical University Taichung City 40447 Taiwan; ^6^ High Performance Materials Institute for x‐Dimensional Printing Asia University Taichung City 41354 Taiwan; ^7^ Division of Pulmonary and Critical Care Medicine Department of Internal Medicine China Medical University Hospital Taichung 40447 Taiwan; ^8^ Graduate Institute of Biomedical Sciences China Medical University Taichung City 406040 Taiwan; ^9^ Department of Laboratory Medicine China Medical University Hospital Taichung City 404332 Taiwan; ^10^ Department of Medical Laboratory Science and Biotechnology China Medical University Taichung City 406040 Taiwan; ^11^ Center for Precision Medicine China Medical University Hospital Taichung City 404332 Taiwan; ^12^ Epigenome Research Center China Medical University Hospital Taichung City 404332 Taiwan; ^13^ H. Milton Stewart School of Industrial and System Engineering Georgia Institute of Technology 755 Ferst Dr NW Atlanta GA 30332 USA; ^14^ School of Materials Science and Engineering Georgia Institute of Technology 771 Ferst Dr NW Atlanta GA 30332 USA; ^15^ Center for Molecular Medicine China Medical University Hospital Taichung City 404332 Taiwan; ^16^ Research Center for Cancer Biology China Medical University Taichung City 406040 Taiwan; ^17^ Department of Neurosurgery China Medical University Hospital Taichung City 404332 Taiwan; ^18^ Translational Cell Therapy Center China Medical University Hospital Taichung City 404332 Taiwan

**Keywords:** decellularized extracellular matrix, lung spheroid model, precision medicine, tumor microenvironment

## Abstract

Lung cancer remains a major health problem despite the considerable research into prevention and treatment methods. Through a deeper understanding of tumors, patient‐specific ex vivo spheroid models with high specificity can be used to accurately investigate the cause, metastasis, and treatment strategies for lung cancer. Biofabricate lung tumors are presented, consisting of patient‐derived tumor spheroids, endothelial cells, and lung decellularized extracellular matrix, which maintain a radial oxygen gradient, as well as biophysicochemical behaviors of the native tumors for precision medicine. It is also demonstrated that the developed lung‐cancer spheroid model reproduces patient responses to chemotherapeutics and targeted therapy in a co‐clinical trial, with 85% accuracy, 86.7% sensitivity, and 80% specificity. RNA sequencing analysis validates that the gene expression in the spheroids replicates that in the patient's primary tumor. This model can be used as an ex vivo predictive model for personalized cancer therapy and to improve the quality of clinical care.

## Graphical abstract

A facile route to construct a highly accurate sophisticated ex vivo lung tumor spheroid model for considering patient‐specific therapies. The LdECM provides native‐tissue‐mimetic physicochemical cues that support the organization of spheroids with a hypoxic signature. The developed patient‐derived tumor spheroid model for the prediction of the optimal chemotherapy or target therapy drug for the patient.

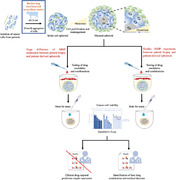

## 1 Introduction

Lung cancer, especially non‐small cell lung cancer, is a leading cause of cancer mortality worldwide owing to its extremely clonal and heterogeneous characteristics.^[^ ^1^ ^]^ Despite the increasing availability of novel therapeutics and genomics analysis, recurrent mutations and subsequent drug resistance have occurred in late‐stage patients, for whom therapeutic selection remains difficult. Therefore, an ex vivo patient‐derived cancer model may play an essential role in filling the gap between genomics and therapeutic responses.^[^ ^2^ ^]^ However, 2D monolayer cell cultures, have been the foundation of new drug developments in cancer research, do not always accurately recapitulate the complex in vivo 3D cellular microenvironments because they lack cell–cell and cell–matrix interactions and the native structures of tumors.^[^ ^3^ ^]^ To realize precision medicine, a study utilized therapeutic selection from a patient‐derived xenograft (PDX).^[^ ^4^, ^5^ ^]^ However, the unacceptable time and cost constraints, in addition to ethical issues related to animal testing, have limited its applicability. Given these limitations, cancer cell‐derived organoids and spheroids are considered desirable systems in cancer research.^[^ ^6^ ^]^ They can provide insight into cancer biology by imitating the pathophysiology of primary tumors and applying it in practical anti‐cancer drug screening.^[^ ^7^ ^]^

Vlachogiannis et al. discovered a strong correlation between primary tissues and patient‐derived organoids in forecasting the treatment outcomes of 21 patients.^[^ ^8^ ^]^ Norman et al. demonstrated that the in vitro responses of breast cancer organoids to tamoxifen highly matched with those of 12 patients, which indicated the potential use of organoids as predictive in vitro surrogates for cancer in vivo.^[^ ^9^ ^]^ Shuford et al. adapted 3D cultured spheroids derived from 44 patients and achieved a high prediction of overall chemotherapy treatment.^[^ ^10^ ^]^ Nevertheless, these systems have several challenges to overcome, including reproducibility and consistency for mass‐production and high‐throughput processes, long culture times (indicating their inadequacy for clinical use), lack of vasculature for oxygen and nutrition exchanged, and absence of validation methods for organoids or spheroids. In addition, considering the complexity of the tumor microenvironment, an in vitro or ex vivo tumor model must include vasculatures that can deliver oxygen and nutrition.^[^ ^11^, ^12^, ^13^ ^]^

**Figure 1:**
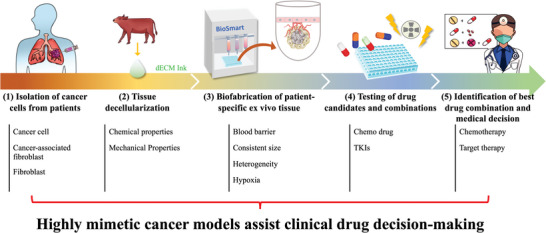
Schematic of the fabrication process and use of the developed patient‐derived tumor spheroid model for the prediction of the optimal chemotherapy or target therapy drug for the patient. Step 1: Lung cancer cells and cancer‐associated fibroblasts are isolated from a biopsy obtained through bronchoscopy. Step 2: Preparation of usable bovine LdECM bioink. Step 3: Patient‐derived cancer cells and cancer‐associated fibroblasts are fabricated using the LdECM bioink to form a patient‐derived lung tumor spheroid model. To mimic the heterogeneous lung tumor ecology, several other matrices are used in the fabrication process, including a vascular cell. Step 4: Patient‐derived tumor spheroid model is cultured for 5 days to recapitulate the pathological features. Then, various panels of chemotherapy or target therapy drug combinations are used on the model. Step 5: Chemotherapy or target therapy drug combinations are prioritized according to their cytotoxicity, and the best combination is determined. Finally, doctors use the assay results to develop a treatment plan for the patient.

The tumor microenvironment comprises complex vasculatures, tissue‐specific extracellular matrix (ECM), immune cells, fibroblasts, and tumor cells.^[^ ^14^ ^]^ Those cells interact and induce ECM remodeling and cellular proliferation, finally influencing cellular behavior and drug delivery. We proposed a novel method as a patient‐specific cancer model to mimic the tumor microenvironment. An *ex vivo* lung cancer model using decellularized ECM (dECM) derived from lung tissues was presented to create a biomimetic tumor microenvironment. Several reports stated that dECM‐regulated cancer cells expressed biomarkers related to metastasis, migration, and mutation.^[^ ^15^ ^]^ **Figure** **1** depicts the fabrication of this *ex vivo* cancer spheroid model. Cancer cells are encapsulated into the lung dECM (LdECM) to form cancer spheroids with vasculature cells to mimic the microenvironment. Gefitinib sensitivity and resistance cell lines, HCC827 and GR10, that are isogenic were adopted to validate the accuracy of this ex vivo model.^[^ ^16^ ^]^ Subsequently, a co‐clinical trial with 20 patients was conducted, and their treatment responses were recorded and compared with the results from ex vivo patient‐specific cancer spheroids. Furthermore, the validated accuracy, sensitivity, and specificity of cancer spheroids were obtained. The basis for developing a novel ex vivo cancer spheroid model was provided and implemented to predict various combinations of drugs. This strategy with LdECM, necrotic core, multi patient‐derived tumor cells, vasculature structure, and quantitative drug efficacy method is novel and may fill the gaps in precision medicine of anti‐cancer drug screening from bench to bed.

## 2 Results and Discussion

### 2.1 Characterization of LdECM Bioink

**Figure 2:**
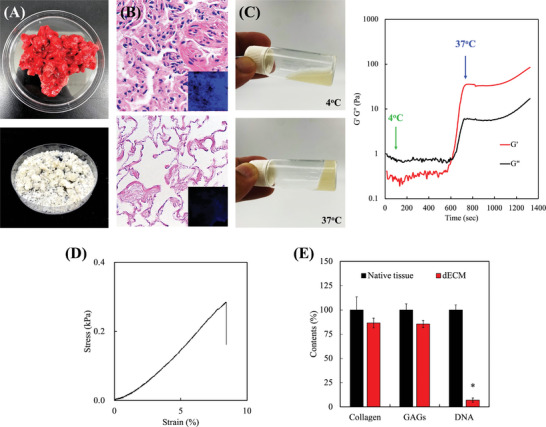
Decellularization of the native lung tissues and biochemical analysis. A) Optical images of native and decellularized lung tissue. B) Representative images of the hematoxylin–eosin (HE) and DAPI staining of native and decellularized lung tissues. C) Photographs captured before and after gelation of the LdECM pre‐gel solution. The rheological properties of the LdECM pre‐gels gelation kinetics from 4 to 37 °C are shown (initial temperature: 4 °C, increment of 5 °C min^−1^ [holding at 15 °C for 5 min], to 37 °C and maintaining at 37 °C for 20 min). D) Representative tensile strain–stress curves of LdECM after gelation for 1 h. E) ECM components (collagen/glycosaminoglycan) and DNA evaluation of native and decellularized lung tissues. All experiments were performed in triplicate, and data are presented as mean ± SEM, *n* = 6 for each group. * indicates a significant difference (*p* < 0.05) from native tissue.

To establish an LdECM bioink, bovine lung tissues were selected for decellularization and sequentially treated with chemical and enzymatic compounds (**Figure** **2**A). Decellularization protocols were evaluated using histological staining and component quantification assays. No cellular remnants were found, although extracellular structures were present (Figure 2B). In contrast to the native lung tissue, the 4′,6‐diamidino‐2‐phenylindole (DAPI) staining of LdECM showed that the nuclei and cell debris were removed, which demonstrated that decellularization functioned as expected. A rheological analysis was performed to determine the sol–gel transition and flow behavior of LdECM, which exhibited a liquid state at 4 °C and behaved similar to a cross‐linked gel at 37 °C (Figure 2C). The properties of LdECM indicated its thermo sensitive sol–gel transition characteristics and that it could be solubilized as a viscous solution at physiological temperatures, making it an excellent material to fabricate and control cell‐laden spheroids easily.^[^ ^17^ ^]^ The storage and loss module results exhibited a similar trend, with the LdECM having a greater storage modulus than the loss modulus at 37 °C. The storage modulus increased after 600 s at ≈15 °C and formed a stable cross‐linked gel at 37 °C after 200 s. Moreover, Young's modulus of 4% LdECM calculated from the strain–stress curve of the tensile test was ≈3.4 kPa (Figure 2D), which indicated that the mechanical properties were identical to those of the native lung tissue.^[^ ^18^ ^]^ The lower concentrations of LdECM had lower Young's moduli than that of the native lung tissue (Figure S1, Supporting Information). Finally, the structural and cellular components of LdECM, such as the collagen and glycosaminoglycans (GAGs) of the tissue and LdECM, were comparable by hydroxyproline and dimethyl methylene blue assays. Yet DNA was virtually non‐detectable in LdECM (Figure 2E). By referring to and altering the decellularization processes from the literature, we established optimal decellularization protocols to effectively eliminate 95% cellular DNA content while preserving the critical components of the ECM, including 80–85% of collagen and GAGs, which were identified by the DNA quantification assay.^[^ ^19^ ^]^ Owing to the component differences between various tissues, their decellularized remains expressed various gelation trends after tissue digestion. Previous studies found that the tissue native microenvironment was the main factor that affected cellular function and fate.^[^ ^20^ ^]^ In this study, an optimal LdECM process and material were developed as a bioink with porous structure and uniformly suspended cells (Figure S2, Supporting Information) for patient tumor cell loading; due to its characterization mimicking naïve lung tissue, it may provide an ideal LdECM for lung tumor cells to express their properties similar to those obtained in vivo.

### 2.2 Engineering of Physiologically Relevant Lung Tumor Models

**Figure 3:**
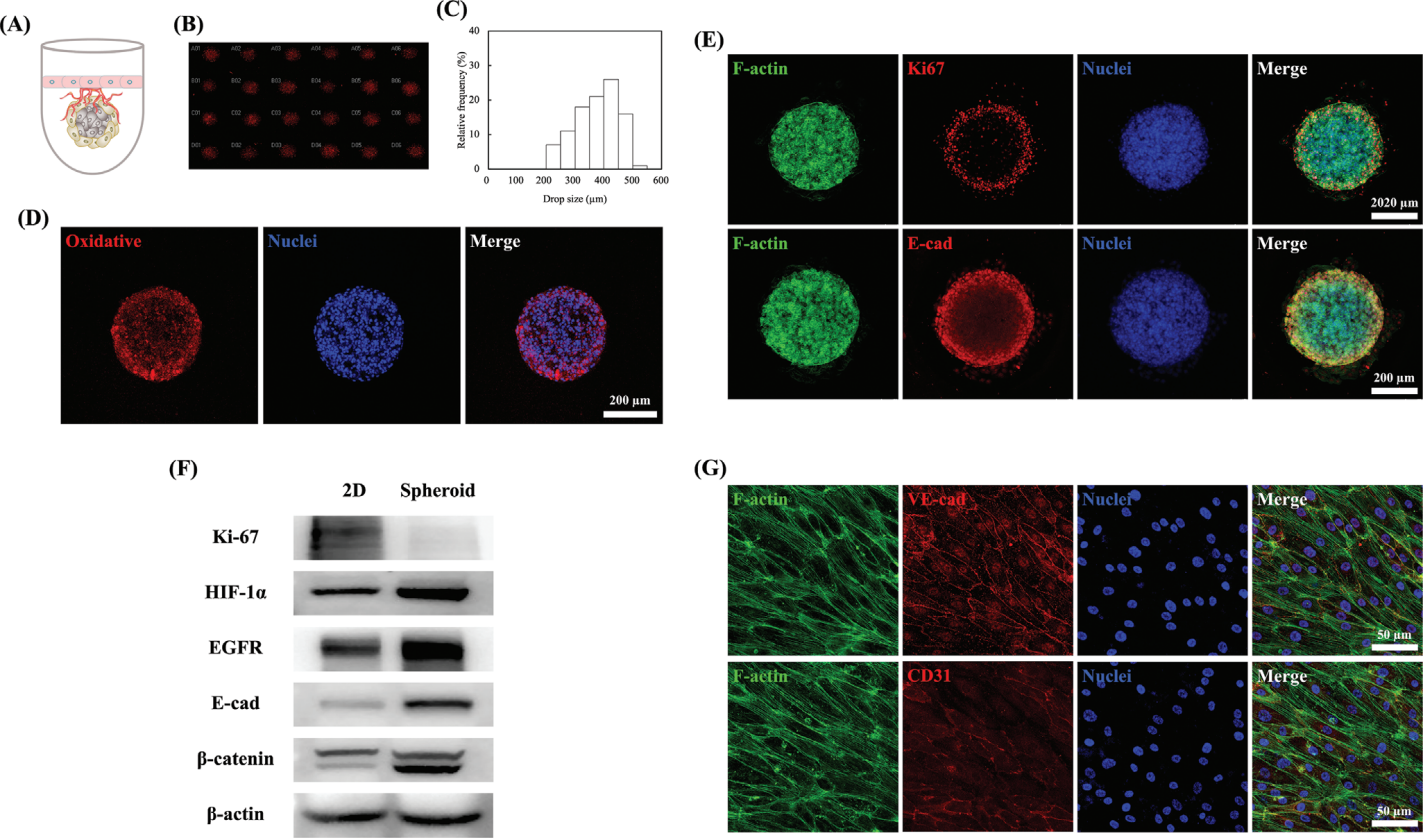
Engineering of lung tumor models where HCC827 spheroids are encapsulated in dECM hydrogels. A) Schematic of patient‐derived lung tumor spheroid model. B) HCC827 spheroids cultured for 2 days in a 96‐well plate. C) Histogram of the spheroid diameters after collecting them from the 96‐well plate (*N* = 60). Staining with D) hypoxia, E) phalloidin‐488 (green), Ki67/E‐Cad rabbit antibody (red), and DAPI (nuclei, blue) of HCC827 spheroids after culturing for 3 days. The scale bar is 200 µm. F) Comparison of the Ki‐67, HIF‐1*α*, EGFR, E‐cad, *β*‐catenin, and *β*‐actin protein expressions of HCC827 for the 2D and spheroid cultured systems for 2 days using western blotting. G) Staining with phalloidin‐488 (green), VE‐Cad/CD31 rabbit antibody (red), and DAPI (nuclei, blue) of HUVEC after culturing for 2 days. The scale bar is 50 µm.

We developed a physiologically relevant 3D in vitro biomimetic model to study lung tumors. Monolayered cell cultures are widely used to study the molecular mechanisms of tumors, particularly the regulation mechanisms of cell proliferation and apoptosis, which are the two most important targets for achieving effective therapy.^[^ ^21^ ^]^ However, using monolayered results to evaluate drug efficacy often underestimates the clinical complexity because the 3D organization of solid tumors, such as cell–cell/cell–matrix interactions and vasculature effects, is overlooked.^[^ ^22^ ^]^ The spheroid models developed in this study can provide an excellent 3D in vitro biomimetic model for simulating hypoxic conditions and native scenarios, thereby facilitating detailed investigations considering drug responses.^[^ ^23^, ^24^ ^]^ This model contains a lung tumor spheroid with functional vasculature and endothelial cells to simulate drug delivery and the blood barrier in vivo (**Figure** **3**A). The number of lung cancer cells in the spheroids were recorded, and the spheroid size histogram was plotted. Finally, a sophisticated and repeatable fabrication process was adopted for fabricating tumor spheroids with various uniform sizes and high cellular viability.

The fabricated spheroids exhibited a consistent morphology (Figure 3B), with ≈65% of the spheres having an average size of 300–400 µm (Figure 3C). These results were consistent with those of previous reports, which indicated that a cell cluster/spheroid diameter range of 400–500 µm was desirable to achieve a necrotic core.^[^ ^25^, ^26^ ^]^ The fluorescent images of the lung tumor spheroids exhibited intact spheroids with a distinctly dark core (Figure 3D). Hypoxic and nutrient‐depleted conditions present in the tumor tissue can be mimicked by these tumor spheroids, indicating that they better reflect the physiological conditions of tumor cell growth in vitro.^[^ ^11^ ^]^ Oxidative regions mainly existed in the outer layer of the cellular spheroid. In tumor tissues, hypoxic gradients develop via the cellular respiration of intervening cells and promote the development of hypoxic regions in cells distal to the oxygen‐supplying blood vessels. Our results indicated that in vitro tumor spheroids cultured under reduced oxygen conditions were strongly hypoxic and activated hypoxia adaptation pathways. Therefore, these in vitro tumor spheroids can provide a suitable system for validating the mechanisms of drug compounds that target tumor hypoxia or anoxia.^[^ ^27^ ^]^ The tumor spheroids stained with E‐cadherin and Ki‐67 (Figure 3E) exhibited tight cell–cell junctions throughout the spheroids, and Ki‐67—a positive proliferative indicator—was detected on their perimeters.^[^ ^28^ ^]^

Combined with the F‐actin and nuclei staining images, live cells with good cell–cell contact were homogeneously distributed within the in vitro spheroids, which is crucial for cell morphogenesis and homeostasis. Given the observation of lung tumor spheroid clustering and characterization of E‐cadherin‐mediated initiation and maintenance of epithelial cell polarity,^[^ ^29^ ^]^ we performed junctional complex protein labeling in the 3D microenvironment system. In Figure 3D,E, the Ki‐67 positive cells are observed to co‐localize with the oxidative results, which indicated that these cells were undergoing proliferation.^[^ ^30^ ^]^ Because the rim of the highly proliferated expression zone was attributed to the optimal exchange of O_2_ and contact with the culture medium, we inferred that the pH in this area of the spheroids was close to neutral, in contrast with the centers of the spheroids, which should exhibit a pH value lower than 7.0 (Figure S3, Supporting Information).^[^ ^31^ ^]^ The Ki‐67, HIF‐1*α*, epidermal growth factor receptor (EGFR), E‐cadherin, and *β*‐catenin levels were further evaluated using western blotting (Figure 3F). The level of Ki‐67 in the spheroid group was lower than that in the 2D culture, validating that the 2D culture exhibited unrestricted proliferation when compared with that in the spheroid group, which was mainly observed at the periphery of the spheroid.^[^ ^32^ ^]^ The high levels of HIF‐1*α* and EGFR in the spheroid group indicated that hypoxic conditions could be replicated using the proposed spheroid model, thereby leading to an increase in the EGFR response to hypoxic conditions.^[^ ^33^ ^]^ In addition, the increments in E‐cadherin and *β*‐catenin levels in the western blot results also indicated the enhanced cell–cell junction formation in this 3D spheroid model.^[^ ^28^ ^]^ In addition, this study considered the bioink cytotoxicity to immune cells since the immune cells play an important role in cancer immunotherapies that should provide the precision prediction in the future. The results showed that THP‐1‐laden LdECM spheroids did not produce cytotoxicity after 2 days of culture (Figure S4, Supporting Information), moreover, we demonstrated the co‐culture images of HCC827, HPF, and THP‐1 in this 3D spheroid (Figure S5, Supporting Information). In the future, the immunotherapy could be evaluated by the further optimal ex vivo model is expected.

Cell–cell interactions are mainly characterized by staining cell junctions and proteins, such as VE‐cadherin, which are also used in confluent monolayer cultures as indicators of the presence of endothelial cells and angiogenesis. The VE‐cadherin and CD31 staining of endothelial cells are illustrated in Figure 3G, indicating that the LdECM developed in this study can upregulate angiogenic‐related genes and markers for angiogenesis. VE‐cadherin and CD31 are responsible for the formation of intercellular junctions between endothelial cells, which is crucial for controlling the diffusion and transport of molecules and endothelial surface polarity.^[^ ^34^ ^]^ Previous studies have demonstrated VE‐cadherin as one of the main endothelial‐specific cell adhesion molecules involved in vascular growth control and morphogenesis.^[^ ^35^ ^]^ Notably, the processing of endothelial cells resulted in the formation of a vascular barrier, which gravitated to the underlying cancer cell spheroids, and a blood‐vessel‐like tissue grew in the cancer cell spheroids (Figure S6, Supporting Information). This ex vivo model with a vascular barrier and blood vessels not only simulates tumor‐induced angiogenesis but also more effectively exhibits the delivery of drugs into the tumor. By fabricating uniformly sized high‐fidelity lung‐tumor spheroids using patient's lung tumor cells to establish a physiologically relevant ex vivo lung tumor model, subsequent drug screening applications may be realized using this system.^[^ ^36^ ^]^

### 2.3 Validation of Drug Sensitivity of Model

**Figure 4:**
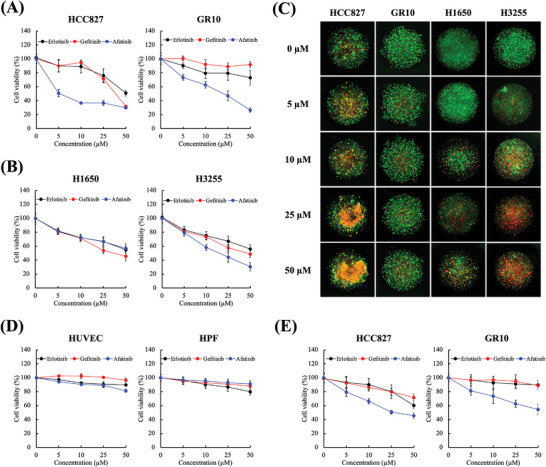
Various cell‐derived spheroids on a model are used to assess the toxicity of targeted drugs. Spheroids are formed using various cell lines: A) HCC827/HCC827 gefitinib‐resistant (GR10), B) H1650/H3255, and C) HUVEC/HPF primary normal cell on model. The model is treated with erlotinib, gefitinib, and afatinib for 2 days. D) Simultaneous use of live/dead images for the analysis of HCC827, GR10, H1650, and H3255 treated with gefitinib. E) Tumors formed by the HCC827/GR10 cells obtained from mice. The isolated cells form spheroids in the model and are treated with erlotinib, gefitinib, and afatinib for 2 days. All experiments were performed in triplicate, and data are presented as mean ± SEM, *n* = 6 for each group.

To validate the lung cancer spheroid model, we examined the cytotoxicity of tyrosine kinase inhibitors (TKIs), erlotinib, gefitinib, and afatinib. The therapeutic mechanism of TKIs is also the most common target therapy that had shown the better effect of patients in the clinic.^[^ ^37^ ^]^ Various lung cancer cell lines, including HCC827, H1650, H3255, and GR10, were cultured with dECM to form spheroids. Subsequently, cellular viability was examined following exposure to TKIs for two days. HCC827 is a lung adenocarcinoma cell line with an acquired mutation in the EGFR‐TK domain (E746‐A750 mutation), and GR10 is a gefitinib‐resistant HCC827 cell line.^[^ ^16^ ^]^ Both H1650 and H3255 are lung cancer cell lines, with no resistance or mutations to first‐generation TKIs;^[^ ^38^ ^]^ whereas H3255 is more sensitive to afatinib.^[^ ^39^ ^]^ The H1650 cells expressed EGFR with exon 19 deletion, whereas the H3255 cells expressed EGFR (L858R), similar to human lung cancer cells with EGFR (L858R) expression,^[^ ^40^ ^]^ thereby making them more sensitive to the EGFR TKIs. As observed in **Figure** **4**A, the three TKIs exhibited concentration‐dependent cytotoxicity on HCC827, with a cytotoxicity of greater than 50%; whereas GR10 required a higher drug concentration to achieve the same level of cytotoxicity. The IC50 values of afatinib for the HCC827 and GR10 cells were approximately 5 and 25 µm after TKI exposure for two days, respectively. Moreover, the cytotoxicity levels of 50‐µm erlotinib against the HCC827 and GR10 cells were 48.9% and 27.0%, respectively. Conversely, an interesting phenomenon was observed in the gefitinib group: A significant difference between the 25‐ and 50‐µm gefitinib with 28.6% and 67.9% cytotoxicity in the HCC827 cells, respectively, whereas gefitinib resulted in only 10% cytotoxicity in the GR10 cells. A similar trend was observed for cytotoxicity results after drug treatment for 5 days (Figure S7, Supporting Information). In addition, the HCC827 cell spheroids treated with various chemotherapeutic drug combinations exhibited a similar trend (Figure S8, Supporting Information). This result clearly indicates that the proposed tumor spheroid model has high specificity and sensitivity for resistant and non‐resistant cell lines.

To further validate the feasibility of this ex vivo lung tumor spheroid model for drug testing, H1650 and H3255 cell spheroids were cultured and evaluated for cytotoxicity. The results revealed that both H1650 and H3255 exhibited similar cytotoxicity trends for erlotinib and gefitinib, compared to the afore described results (Figure 4B). At 10‐µm afatinib, a significant difference between the cytotoxicity levels of H3255 (41.7%) and H1650 (27.5%) and concentration‐dependent cytotoxicity was observed for afatinib in all cell lines. Figure 4C displays the live/dead images of these four cell lines; all indicate similar live/dead trends when treated with gefitinib in a concentration‐dependent manner. In addition to the lung cancer cell lines, we utilized non‐tumor cells of human umbilical vein endothelial cells (HUVEC) and human pulmonary fibroblasts (HPF) to validate the specificity of this spheroid model (Figure 4D).

After validating the developed spheroid model using cell lines, an advanced validation system of cell‐line‐derived xenograft (CDX) with HCC827 and GR10 cells implanted in NOD‐SCID‐gamma (NSG) mice was used for tumorigenesis. We have designed the experimental protocol to harvest mice tumors when their volume exceeded 1000 mm^3^, isolated the tumor‐related cells, and fabricated tumor spheroids for TKI testing. As depicted in Figure 4E, the isolated tumor cells from the HCC827‐derived tumors are sensitive to the three TKIs, whereas the GR10‐derived tumor spheroid is insensitive to gefitinib. In the GR10 group, erlotinib and gefitinib had minimal cytotoxic effects on the GR10‐derived tumors, whereas afatinib had concentration‐dependent cytotoxic effects. Notably, the CDX‐derived spheroid models had lower cytotoxicity levels than those of the primary cancer cell lines (Table S1, Supporting Information). We deduced that multi‐cell types, such as cancer‐associated fibroblasts (CAFs), immune cells, and interstitial cells, coexisted with cancer cells in the in vivo tumor microenvironment, which was the main mechanism resulting in the sensitivity difference between the spheroid models of the derived primary cell line and that of the CDXs.^[^ ^41^ ^]^ The lung cancer spheroid model recapitulates resistant and sensitive phenotypes of the known cell lines, thus, calls for further testing human samples.

### 2.4 Analysis of Patient Treatment Responses

**Figure 5:**
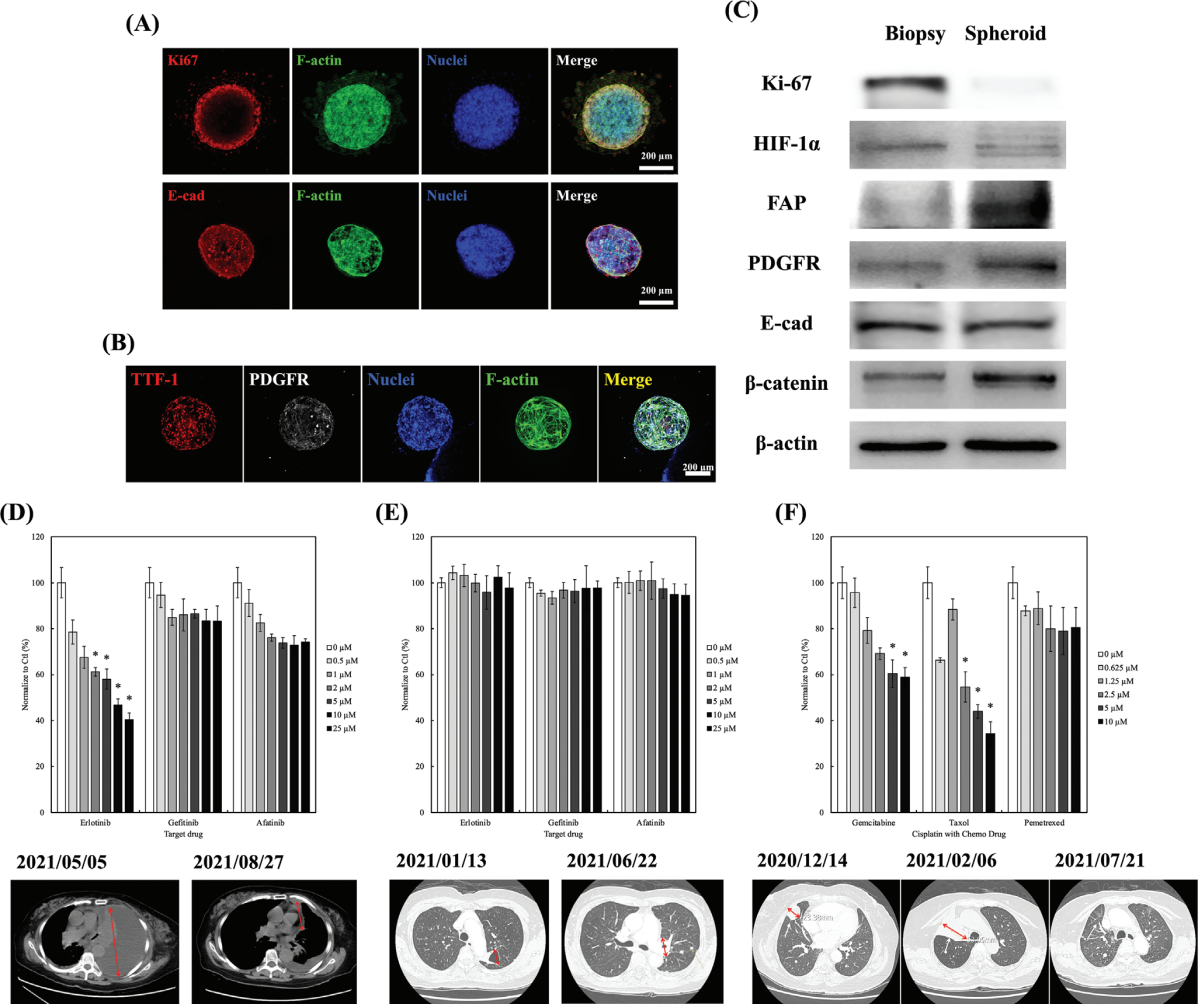
Characterization of primary lung‐cancer spheroids. A) Ki67/E‐Cad rabbit antibody (red), B) TFF‐1 rabbit antibody (red), PDGFR mouse antibody (gray), phalloidin‐488 (green), and DAPI (nuclei, blue) for patient primary tumor‐derived spheroids after culturing for 3 days. The scale bar is 200 µm. C) Comparison of Ki‐67, HIF‐1*α*, EGFR, E‐cad, *β*‐catenin, and *β*‐actin protein expressions of the biopsy and spheroid tests by western blotting. D,E) Responses of lung tumor spheroids from various patients to erlotinib, gefitinib, and afatinib (*n* = 3, data are presented as mean ± SD). The CT scans indicate that the pretreatment images (left) show the mass lesions of each patient. The final follow‐up images (right) show d) decreased mass lesions, and E) multiple enhanced mass lesions. F) In addition, one patient showed a largely enhanced mass lesion after treatment for 2 months. This patient‐tumor‐derived spheroid model, which utilizes gemcitabine, is shown to be therapeutically effective. The tumor shrinks significantly after another 4 months of treatment with gemcitabine. * indicates a significant lower (*p* < 0.05) than 70%.

A co‐clinical investigation spanning 20 patients was conducted to elucidate the prediction accuracy of the developed patient‐derived tumor spheroid model. The preclinical spheroid results were shared in real time to visualize drug response and resistance data for the patients in this clinical study. In the clinical trial protocol, we performed a computed tomography (CT)‐guided tissue biopsy on patients to elicit the pathological proof of primary lung tumors. The primary lung tumor‐related cells were isolated and formed to spheroids with LdECM. Cells isolated from the biopsy tissue were identified using immunofluorescence staining for specific lung cancer biomarkers (EpCAM).^[^ ^42^ ^]^ Approximately 76% of the cells exhibited EpCAM and were presumed to be lung cancer cells; the others were assumed to be CAFs or stromal cells (Figure S9, Supporting Information) from patient's biopsy samples. The fluorescent staining results showed that cells near the periphery of the spheroids had higher expression levels of Ki‐67, and majority of the cells exhibited E‐cadherin protein expression, which indicates that the laden cells in the spheroids were mainly cancerous (**Figure** **5**A). Furthermore, TTF‐1, a biomarker used as a diagnostic marker for lung adenocarcinoma and small‐cell carcinoma, was observed in the ex vivo tumor spheroids (Figure 5B).^[^ ^43^ ^]^ In addition to cancer cells, CAFs in native tumors are a critical factor in lowering the efficacy of anti‐cancer drugs. PDGER expression, which is a specific protein marker commonly used to determine the differentiation and metastasis of CAFs, was observed in the patient‐tumor‐derived spheroids.^[^ ^44^ ^]^ The cancer cell‐ and CAF‐related biomarkers expressed in the patient‐derived lung tumor spheroids after culturing resembled those expressed in the primary tumor tissues (Figure 5C). This proves that the proposed spheroid model is not only structurally similar to tumor tissues but also exhibits tumor heterogeneity. The investigated compositions significantly affect the accuracy and precision of drug treatment planning.^[^ ^45^ ^]^

**Figure 6:**
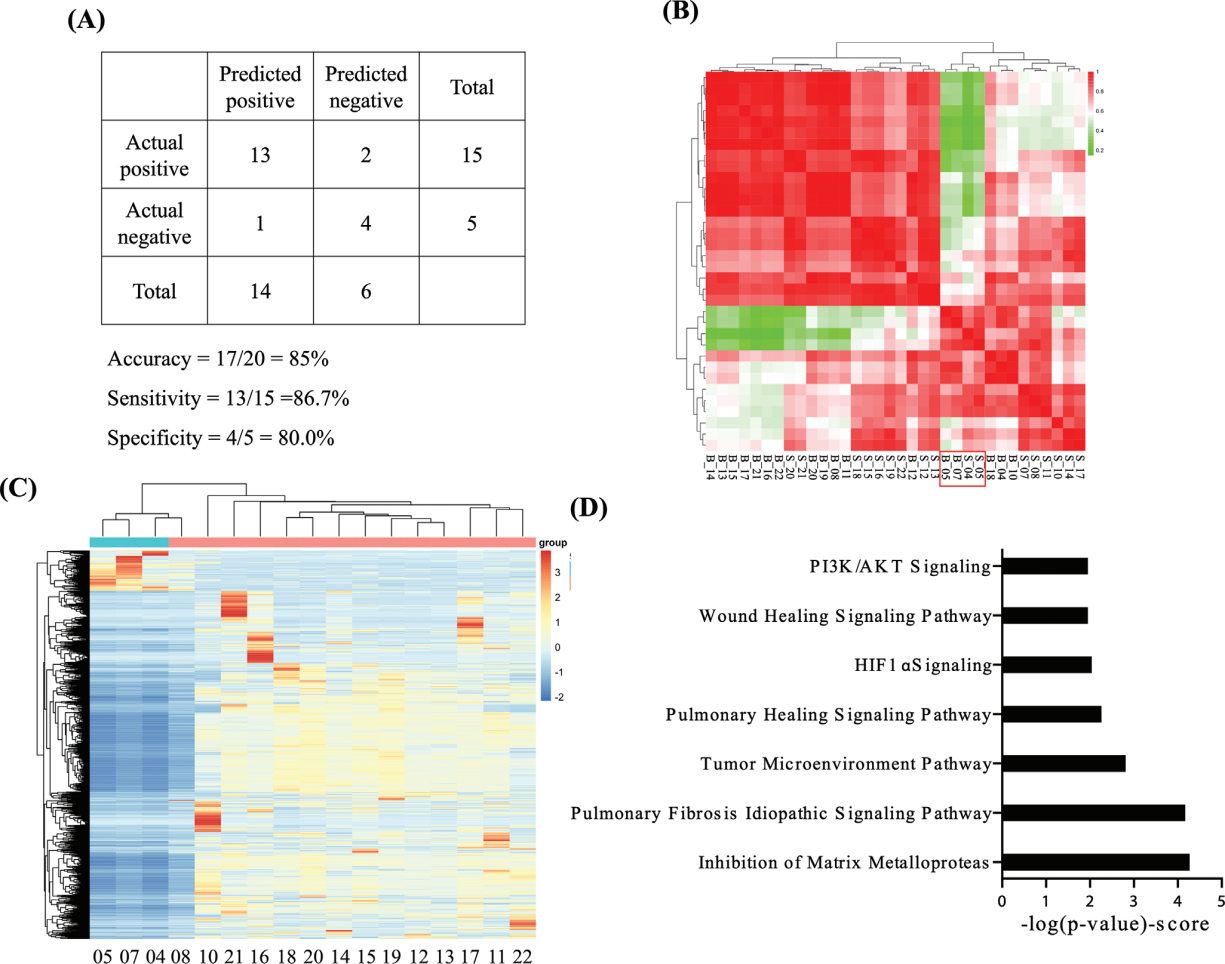
Characterization of primary lung‐cancer spheroids. A) Correlation and predictive data from model analysis based on patient post‐treatment results. B) Spearman correlation matrix revealing strong correlations between the primary tumor tissue and patient primary tumor‐derived spheroids. C) Differentially expressed genes in D) the top seven canonical pathways derived from genes in the leading edge, analyzed using IPA pathways (*p* < 0.001). The statistically significant pathways identified are listed according to their *p*‐value (−log).

Based on the protocols (CMUH109‐REC2‐124), patient tumors were isolated, formed into spheroids, and exposed to TKIs for two days. Figure 5D,E displays the test results from two patient‐tumor‐derived spheroids and their TKI clinical outcomes. Case in Figure 5D was randomly selected from drug sensitive groups in both spheroid model and clinical results. Figure 5D illustrates the drug responses of this patient's tumor‐derived ex vivo spheroids, indicating that erlotinib had concentration‐dependent cytotoxicity, with a cell viability of 40.4% at 25 µm. However, the test results of gefitinib and afatinib on the same patient demonstrated a high cell viability of more than 75%, with no concentration‐dependent cytotoxicity. Therefore, we inferred that this patient exhibited no response to gefitinib and afatinib, which was considered in the treatment strategy for the patient. After six months of treatment with erlotinib, as indicated in Figure 5D, this patient's tumor shrunk significantly, and erlotinib demonstrated a partial response to the lung cancer in this patient. In contrast, Figure 5E demonstrates another case randomly selected from the drug resistant groups in both spheroid model and clinical results. It exhibited no response to the three TKIs (cell viability greater than 90%) from the spheroid testing results. Based on clinical guidelines, the patient was advised to undergo erlotinib treatment for six months. The follow‐up CT images indicated that erlotinib could not restrict the disease's progress, and the tumor size significantly increased. This result is consistent with the efficacy prediction of patient‐derived spheroids. Finally, this co‐clinical investigation of the proposed patient‐tumor‐derived spheroid model demonstrated high accuracy (85%), sensitivity (86.7%), and specificity (80%) (**Figure** **6**A). Although most patients in this study received targeted therapy, encouraging results were obtained for patients who were administered chemotherapy. In one patient, the tumor gradually grew after pemetrexed medication for two months, thus this medication was considered an ineffective prognosis. The oncologist subsequently made a challenging decision to switch the gemcitabine medication strategy based on spheroid model result. After gemcitabine administration for another 4 months, this patient's tumors remarkably shrank. Our model is not only proved to have high prediction rate to patients with EGFR mutation but also has high potential to predict the chemo‐drug efficacy for patient with wild‐type EGFR.

### 2.5 Role of Matrix Metalloproteases in Altering Cancer Microenvironment

In order to have deeper understanding of this accuracy of ex‐vivo spheroid model, we dissected the inconsistent drug responses of three patients (LC04, LC05, and LC07) between their clinical outcomes and preclinical spheroid tests. The gene expression profiles with a correlation matrix revealed strong correlation between the primary tumor tissue and patient‐derived spheroids (Figure 6B). Figure 6C illustrates a heat map of the differential expression analyses for the treatment‐responsive spheroid samples. The evaluation of biological function using ingenuity pathway analysis (IPA) showed a significant enrichment in the inhibition of matrix metalloproteases (MMP; −log(*p*‐value) = 4.27), pulmonary fibrosis idiopathic signaling pathway (−log(*p*‐value) = 4.17), tumor microenvironment pathway (−log(*p*‐value) = 2.81), pulmonary healing signaling pathway (−log(*p*‐value) = 2.26), HIF1*α* signaling (−log(*p*‐value) = 2.04), wound healing signaling pathway (−log(*p*‐value) = 2.02), and PI3K/AKT signaling (−log(*p*‐value) = 2.02) (Figure 6D). In addition, several pathways were enriched in the lung tissue microenvironment system, including angiogenesis and those related to matrix regulation, and the cellular community. Among them, the HIF1‐*α* signaling pathway is critical for cancer cell growth and metastasis. Interestingly, several MMP genes from the inconsistent groups were strongly expressed in the inconsistent biopsy groups but not in the patient tumor‐derived spheroids. The tumor microenvironment signaling pathway and inhibition of the MMP signaling pathway are involved in the modulation of cancer progression responses. We performed clustering for drug treatment samples based on gene expression profiles using k‐means clustering for drug response prediction. Despite the biological differences between the drug response groups, most drugs targeting the LC08 specimens clustered similar to the inconsistent groups, indicating the inhibition of the MMP pathway, thereby resulting in the difference in these test results. Several studies over the past two decades have identified MMP as a mediator of several important cancer processes.^[^ ^46^, ^47^ ^]^ Therefore, we believe that MMP plays a key role in changing the tumor microenvironment in ex vivo patient‐derived spheroids, causing inconsistent testing results.

## 3 Conclusions

This study established a facile route to construct a sophisticated *ex vivo* lung tumor spheroid model with essential factors in terms of ECM components, cell phenotypes, and vascular barriers for considering patient‐specific therapies. The composition and physicochemical properties of bovine LdECM provided native‐tissue‐mimetic physicochemical cues that support the organization of spheroids with a hypoxic signature from either cell lines or primary tumor cells, as well as a vasculature barrier from HUVEC, without altering their susceptibility to various anti‐cancer drugs. Further, the excellent prediction results of co‐clinical trials (accuracy: 85%; sensitivity: 86.7%; specificity: 80%) and RNA sequencing analysis confirm the success of the lung cancer preclinical spheroid model. Particularly, this preclinical examination can be used in the point‐of‐care analysis because the timeline is within 12 days. We expect that this strategy should help to develop various disease or tumor models and ultimately guide clinical decisions.

## 4 Experimental Section

### Preparation and Characterization of the LdECM Pre‐Gel Solution

The decellularized extracellular matrix used in this study were extracted from the lungs of bovine. The harvested tissues were obtained from animals of completed studies and the original study is completely unrelated to the current study by the Animal Experimental Ethics Committee of China Medical University (CMUIACUC‐2021‐151). A total of 40 organs were expected to be collected each year for analysis. After slicing the tissues into 0.1 cm slices, the tissues were first washed with phosphate buffered saline (PBS) buffer solution to remove blood components and further mixed and react with 0.1% Triton‐X100 solution at room temperature for 30 min to cause cell lysis. PBS was then used to wash the tissues and stirred overnight in a 4 °C SDC solution. The solution was then changed to deionized water and stirred for at least another 2 h. After which, the tissues were stirred in 1 m NaCl aqueous solution and for 30 min, washed with deionized water for 30 min, and stirred again with 1% Triton x 100 solution for 30 min. The above procedures were done to cause repeated cell lysis and removal of cellular components. The tissues were then washed with ionized water and kept in a −80 °C freezer for 24 h before undergoing lyophilization at −20 °C to obtain the LdECM.

In order to consider the efficacy of the decellularization processing, the histological sections were analyzed with the Hematoxylin and Eosin (HE) and 4′,6‐diamidino‐2‐phenylindole (DAPI) staining, and the residual DNA, Col, GAGs were quantitative assessed as well. The images of sections were observed using a fluorescent microscope (OLYMPUS BX53) to determine if our decellularization was complete. To prepare LdECM solutions, LdECM samples were sterilized in 75% ethanol for 2 h, then rinsed thoroughly with PBS, cut into small pieces and digested in 1 mg mL^−1^ pepsin (CALZYME Laboratories) in 0.5 n acetic acid solution to obtain various concentrations of LdECM. Then, the mixture was stirred to fully dissolve the dECM. A 100 µm filter was used to filter out any residues that have not been completely dissolved, and the solution was stirred at 4 °C while performing acid‐base titration with 1 n NaOH titration till pH 7.4. A rheology analyzer (MCR92 Anton Paar) was used to measure the viscoelasticity of dECM at a temperature range from 4 to 37 °C, at an increment rate of 1 °C per minute for 1 h. In addition, the LdECM bioink was formed a dumbbell‐shaped tensile sample with a thickness of 2 mm prepared using a mold and casting method in accordance with ISO specifications. Afterward, the sample prepared by using LdECM would be placed with the mold in a 37‐degree incubator for 1 h. The temperature would allow the LdECM to gelation and cross‐linking processing. The standard sample piece was analyzed using a table‐top tensile tester (EZ‐Test machine, Shimadzu, Kyoto, Japan). The tensile test was conducted at a speed of 0.2 mm min^−1^ until each sample was completely torn apart. The yield strength, strain, and Young's modulus levels of each group were measured and transformed into several graphs according to the recorded results. At least six samples of each group were analyzed and the average was recorded. The efficiency of decellularization was determined by quantitative measurements of DNA, glycosaminoglycans (GAGs), and collagens contents in both native and decellularized tissues with specific kits. Briefly, the DNA contents of tissues were performed by using the lysate obtained by using the DNeasy Blood and Tissue kit (Qiagen, Germany) according to the manufacturer's instructions. The minced tissues were lysed with a protease K solution at 56 °C and the total DNA was purified. The purity of the extracted DNA was confirmed by using a spectrometer to measure the ratio between absorbances at 260 and 280 nm. The DNA contents of the samples were quantified with a Qubit Fluorometer (ThermoFisher, USA). Dimethylmethylene blue (DMB) assay was employed to analyze the GAGs contents of tissues. The minced tissues were digested in a phosphate buffer solution (PBS) supplemented with 125 µg mL^−1^ papain, 10 mm cysteine, and 10 mm EDTA at 60 °C for 18 h. Afterward, the supernatant was reacted with DMB dye, and the absorbance at 525 nm of the mixture was read by a spectrometer. For the collagens content, the minced tissues were incubated with a HCl solution containing pepsin at 4 °C overnight. The collagens contents of the soluble products were measured by using a Sircol Assay kit (Biocolor Life Science, UK) according to the manufacturer's instructions. The absorbance at 555 nm of the reactant solution was measured on a spectrometer and the collagens content was calculated.

### 3D Lung Spheroids Model

All cells were cultured in their respective appropriate medium. The culture medium was changed every 2 or 3 days. Prior to the LdECM‐embedding culture, each cell type was harvested by treating with a solution of 0.25% trypsin in EDTA (Gibco), collected the cells by centrifugation at 1200 rpm. for 10 min and then dispersed the cells in each culture medium. The cell suspensions were mixed with the pre‐gel solutions (4 wt%) of LdECM to a final concentration of 1 × 10^7^ cells mL^−1^ for various cells. The whole process for the engineering of physiologically relevant lung tumor models were printed by the BioSmart Ver.1.0 (Figure S10, Supporting Information). 27G printing needle was used, the printing temperature was set to 4 °C, and the working pressure was 15–20 kPa. the printed procedure was carried out in a 96‐well at a nozzle moving speed of 20 mm s^−1^ between each droplet. The volume of pre‐gel solution for each well was set to 0.5 µL (5000 cells per spheroid). Subsequently, the pre‐gel solutions (2 wt%) of LdECM without cells were gel‐filled 50 µL and inserted into the well by the second cartridge followed by being placed in the incubator at 37 °C for 1 h. After gelation, the culture medium with endothelial cells was added to the wells by the third cartridge. Each culture medium was changed every 2 or 3 days.

### pH and Oxygen Concentration Analysis

The commercially available dyes SNARF‐1 AM (C1272, Thermo Fisher) and Image‐It hypoxic reagent (H10498, Thermo Fisher) were used to analyze distribution of pH and oxygen gradients during cultured. To measure pH, the spheroids were reacted with 10 *µ* m SNARF‐1 AM for 10 min, and then washed twice with PBS. The pH‐related images of spheroids were observed at 488 nm and 594 nm wavelength. In addition, the hypoxic reagent was used to analyze the oxygen distribution and gradients. For this test, the fluorescence increased with a decrease in oxygen concentration. 10 *µ* m of the hypoxic reagent was added for 24 h and observed with 488 nm/650 nm wavelength.

### Immunofluorescence Staining

Tumor spheroids in response to biomarkers expression were visualized by immunofluorescence. After spheroids generation, the spheroids were washed with phosphate buffer solution (PBS), then immersed in 4% formaldehyde at room temperature for cell fixation and permeabilized with 0.2% Triton X‐100 in PBS for 1 h. The spheroids were then reacted with primary antibodies such as anti‐Ki67 (ab16667, Abcam), anti‐E‐cadherin (ab40772, Abcam), anti‐TTF1 (thyroid transcription factor, ab204411, Abcam), anti‐PDGFR (platelet‐derived growth factor receptor, ab69506; Abcam) for tumor spheroids and anti‐CD31 (ab24590, Abcam), and VE‐cadherin (ab33168, Abcam) for HUVEC that were performed overnight at 4 °C, followed by rinsing three times for the constructs with PBS. Then, Alexa 594 secondary antibody (Invitrogen, Carlsbad, CA, USA), phalloidin‐Alexa 488 (Invitrogen) were then incubated in the dark for 3 h, and DAPI (Invitrogen) for 30 min. Lastly, the constructs were rinsed with PBS and followed by imaging samples with a confocal microscope (Dragonfly Spinning Disc Confocal Microscope, Oxford Instruments Andor, CT, USA).

### Western Blot

The cellular lysates were prepared according to the authors’ previous instructions. Briefly, total protein lysates were obtained via NP40 lysis buffer. The protein concentration was detected by BCA assay (Bio‐Rad). Proteins were resolved under 10% SDS‐PAGE conditions, then transferred to polyvinylidene difluoride blotting membranes (PVDF) membranes (Immobilon, Bedford, MA). The membranes were blocked with 5% BSA for 1 h at room temperature, then incubated with primary antibody Ki67 (ab16667, Abcam), HIF‐1*α* (ab179483, Abcam), EGFR (A11351, Abclonal), Anti‐Fibroblast activation protein (FAP, ab28244, Abcam), PDGFR (ab16667, Abcam), E‐cadherin (ab40772, Abcam), *β*‐catenin (ab16051, Abcam), and *β*‐actin (A5441, Sigma‐Aldrich, St. Louis, MO, USA) at 4 °C overnight. After three washes with TBST (20 mm Tris, 150 mm NaCl, 0.1% w/v Tween‐20), the membranes were subsequently incubated with the horseradish peroxidase‐conjugated secondary antibodies for 1 h at room temperature. Finally, three washes with TBST and visualized by enhanced chemiluminescence using ImageQuant LAS 4000 (GE Healthcare, Pewaukee, WI).

### Drug Response

In order to confirm the accuracy of the lung cancer model, the first‐ and second‐generation targeted tyrosine kinase inhibitors (TKIs) drugs used in the clinic for testing, such as erlotinib, gefitinib, and afatinib, were directly used. After fabricated the lung spheroids model, the TKIs were used to treat for another 2 days. To consider cell viability of spheroids in the lung cancer model by PrestoBlue HS (Invitrogen) as described above. In brief, the PrestoBlue HS was added into the medium at a ratio of 1:9 and placed in the incubator for 2 h for reaction, after which the solution was transferred to new 96‐well microplate. A spectrophotometer (Infinite Pro M200, Tecan, Männedorf, Switzerland) was used to read the fluorescence and Excitation/Emission was 560/590 nm. All experiments were performed in triplicate and the average was recorded. In order to observe the survival of spheroids encapsulated in LdECM, Live & Dead analysis was done and the spheroids were observed with a confocal microscope (Leica TCS SP8, Wetzlar, Germany). With the Live & Dead kit, live cells were stained with green fluorescent calcein acetoxymethyl (Calcein AM) and dead cells were stained with red fluorescent Ethidium homodimer‐1 (EthD‐1) in order to distinguish between live and dead cells.

### HCC827 and GR10 Orthotopic Xenograft Mouse Models

The orthotopic xenograft mouse model experimental procedures employed in the present study were approved by the Animal Experimental Ethics Committee of China Medical University (CMUIACUC‐2020‐268). Six to eight weeks old NOD/SCID gamma (NSG) mice were purchased from BioLASCO. Male mice were used for the xenograft lung tumor model. HCC827 and GR10 cells were resuspended in PBS containing Matrigel, then the cells (1 × 10^6^ per 100 µL) were injected subcutaneously. After the injection, all mice were allowed to rest for at least 30 min before the wound was closed. After implantation, the mice were sacrificed and harvested the tumors when tumors reached 150–200 mm^3^. Tumor volumes were calculated using the following equation: Tumor volume = (length × width × width)/2. The surgical specimens were split into three different regions for spheroids preparation. For tissue dissociation, the fresh tissues were cut into 1–5 mm^3^ pieces and digested in dispase (2 U mL^−1^) and collagenase (1 mg mL^−1^) on an orbital shaker at 37 °C. The cell suspension was passed through the glass pipette and 40 µm sieve to generate a single‐cell suspension. These cells were centrifugated at 1500 rpm for 5 min and the cell pellet was re‐suspended in 6‐cm dish with DMEM (Invitrogen). Subsequently, these cells were prepared into a lung tumor spheroids model according to the previous method, and the cytotoxicity was analyzed using TKIs.

### Clinical Outcome Evaluation

Lung cancer and normal‐adjacent specimens were obtained from China Medical University Hospital. This study was assessed and approved by the ethics committees of each respective institute (CMUH109‐REC2‐124). Informed approval was received prior to the acquisition of specimens from all donors. The surgical specimens were split into three different regions for spheroids preparation, histology assay, and gene sequencing. For tissue dissociation, the fresh tissues were cut into 1–5 mm^3^ pieces and digested in dispase (2 U mL^−1^) and collagenase (1 mg mL^−1^) on an orbital shaker at 37 °C. The cell suspension was passed through the glass pipette and 40 µm sieve to generate a single‐cell suspension. These cells were centrifugated at 1500 rpm for 5 min and the cell pellet was re‐suspended in 6‐cm dish with Advanced DMEM (Invitrogen). After several days of culture, some cells were subjected to fluorescent staining of lung cancer biomarkers (EpCAM, A19301, ABclonal) and nuclei to identify the proportion of cancer cells in all isolated cells. Subsequently, these cells were prepared into a lung tumor spheroids model according to the previous method, and the cytotoxicity was analyzed using a variety of drug combinations. After few months, the evaluation of the patient's treatment effect would be performed by computed tomography (CT), and would be classified by the attending physician according to Response Evaluation Criteria in Solid Tumors (RECIST version 1.1), which could be divided into complete response (CR), partial response (PR), stable disease (SD), and progressive disease (PD). CR and PR revealed that the drug responded well to the patient. For patients with locally advanced or metastatic lung cancer that could not be cured, the main purpose of clinical treatment was to improve survival and maintain quality of life, and the outcome of SD was acceptable.

### RNA Sequencing

Total RNA was extracted from clinical tissue samples, tissue‐derived cells, and tissue‐derived spheroid using a NucleoSpin RNA Kit (Macherey‐Nagel GmbH, Düren, Germany) according to the manufacturer's instructions. The quality, quantity, and integrity of the extracted RNA were evaluated using a NanoDrop1000 spectrophotometer and an Agilent 2100 Bioanalyzer (Agilent Technologies, Santa Clara, CA, USA). Samples with RNA integrity (RIN) >8.0 were used for RNA‐seq. An mRNA‐focused, barcoded library was generated using a TruSeq Stranded mRNA Library Preparation Kit (Illumina, San Diego, CA, USA). The libraries were sequenced using the Illumina Nova Seq 6000 platform (Illumina), using 2 × 151 bp paired‐end sequencing flow cells according to the manufacturer's instructions.

### RNA‐Seq Data Analysis

The RNA‐seq data were analyzed as described previously.^[^ ^2^ ^]^ In brief, data quality control at the Q20 level was performed using Trimmomatic, read alignment to the GRCh38 human genome was conducted using HISAT2, and expression was quantified using GENCODE v22 (excluding mitochondrial genes), and transcripts were normalized into transcripts per million (TPM) using StringTie.

### Functional Enrichment Analysis

Differential expression genes were determined with 2× fold change compared with LC04, LC05, LC07 group (whether is upregulated or downregulated) The molecular relationships were generated using the Core Analysis showing significant (*p* < 0.05) association by Ingenuity Pathway Analysis software (QIAGEN company, Redwood City, CA, USA). Canonical pathways analysis found by core analysis in IPA are given with a *p*‐value.

### Statistical Analysis

A one‐way statistical analysis of variance (ANOVA) was applied to analyze the significance of the difference between the different experimental groups in each experiment. Determination of a significant deviation in each sample was carried out using Scheffe's multiple comparison test. A *p*‐value <0.05 was considered statistically significant, as indicated by “*” in the different group comparisons.

## Conflict of Interest

The authors declare no conflict of interest.

## Supporting information

Supporting Information

## Data Availability

Research data are not shared.
