# Supplementary material for: Highly Mimetic Ex Vivo Lung‐Cancer Spheroid‐Based Physiological Model for Clinical Precision Therapeutics
Source: Adv Sci (Weinh). 2023 Apr 21;10(16):2206603. doi: 10.1002/advs.202206603 (PMC10238206; doi:10.1002/advs.202206603)
Supplement: Supplementary file 1 — Supporting Information [file ADVS-10-2206603-s001.pdf]

ADVANCED SCIENCE
Open Access

Supporting Information

for *Adv. Sci.*, DOI 10.1002/advs.202206603

Highly Mimetic Ex Vivo Lung-Cancer Spheroid-Based Physiological Model for Clinical Precision Therapeutics

Ming-You Shie, Hsin-Yuan Fang, Kai-Wen Kan, Chia-Che Ho, Chih-Yen Tu, Pei-Chih Lee, Po-Ren Hsueh, Chia-Hung Chen, Alvin Kai-Xing Lee, Ni Tien, Jian-Xun Chen, Yu-Cheng Shen, Jan-Gowth Chang, Yu-Fang Shen, Ting-Ju Lin, Ben Wang, Mien-Chie Hung, Der-Yang Cho* and Yi-Wen Chen**

Table S1. The IC_{50} of various cells treated with targeted therapy drug for 48 h.

	Erlotinib (μM)	Gefitinib (μM)	Afatinib (μM)
HCC827	51.37	38.47	18.93
GR10	96.40	276.55	27.47
H3255	52.69	42.59	28.25
H1650	51.79	39.04	54.36
HCC827(Xenograft)	63.94	89.24	37.43
GR10(Xenograft)	270.72	241.06	49.57
HUVEC	261.99	537.45	146.82
HPF	127.60	236.28	311.14

Table S2. Clinical details of patient's information, diagnostic information and treatment medication list.

Code	Age	Sex (0=W; 1=M)	Height	Weight	Smoke(0=N; 1=Y)	Diagnosis	Pathology	EGFR	Treatment	Drug
LC001	60	0	154.4	73.2	0	201013	SqCC	L858R	201105	Erlotinib
LC002	61	0	152	60	0	201223	Adenoacarcinoma	L858R	210115	Erlotinib
LC003	80	0	159	58.5	0	201229	Adenoacarcinoma	L858R	210115	Erlotinib
LC004	74	1	170	63.5	0	201231	Adenoacarcinoma	deletions in exon 19	210115	Osimertinib
LC005	55	1	168.5	78.5	1	201230	Adenoacarcinoma	-	210121	Alectinib
LC006	62	0	147.6	56.9	0	201225	Sarcomatoid carcinoma	-	210112	Carboplatin+Alimta
LC008	58	1	167	67.5	1	210225	Adenoacarcinoma	letions in exon 19, T79(	210727	Osimertinib
LC010	67	0	163.9	54.9	0	210329	Adenoacarcinoma	L858R	210407	Erlotinib
LC011	80	1	170.4	58.2	0	200228	Adenoacarcinoma	-	210314	Alimta+Carboplatin
LC012	59	0	158	45	0	210427	Adenoacarcinoma	deletions in exon 20	210430	Osimertinib
LC015	55	0	152.7	60.1	0	210511	Adenocarcinoma	L858R	210521	Erlotinib
LC016	57	1	170.7	79.2	0	210521	Adenoacarcinoma	L858R	210526	Afatinib
LC018	68	0	148.5	43.9	0	210708	Adenocarcinoma	etions in exon 19, Exon	210716	Erlotinib
LC021	53	1	155	64.1	1	210816	Adenocarcinoma	deletions in exon 19	210908	Osimertinib
LC022	69	1	160.9	60.1	1	210521	Adenoacarcinoma	-		Pemetrexed
LC023	53	0	164.4	56.1	0	210320	Adenocarcinoma	deletions in exon 19	210407	Erlotinib
LC025	71	0	149.2	61.7	0	201218	Adenocarcinoma	letions in exon 19, T79(	210825	Osimertinib
LC026	56	0	160.5	63.7	0	210318	Adenocarcinoma	deletions in exon 19	220416	Erlotinib
LC027	62	1	165	60.4	1	210128	Adenoacarcinoma	deletions in exon 20	220210	Afatinib
LC028	62	0	154.4	57	0	210408	Adenoacarcinoma	-	210427	Cisplatin+Alimta

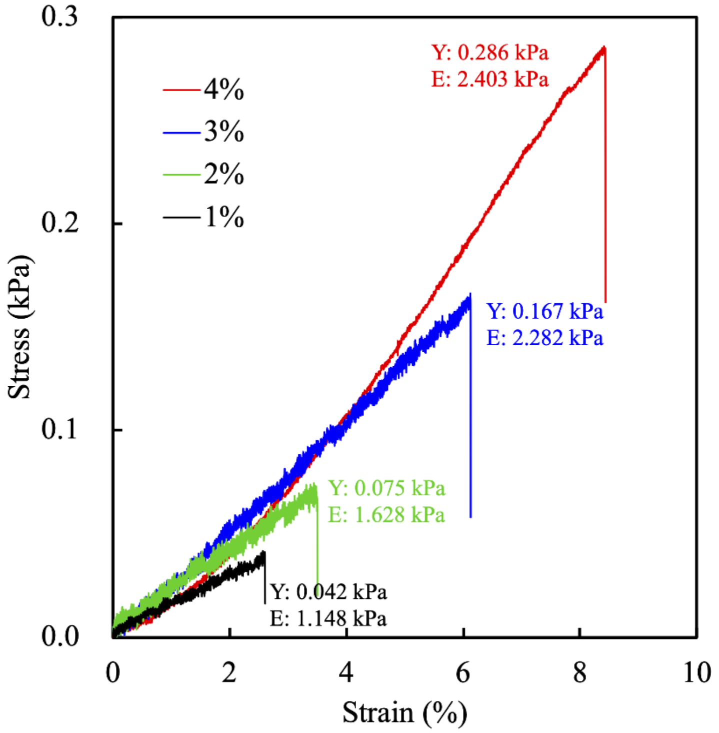

Figure S1. Representative tensile strain–stress curves for various concentrations of LdECM after gelation for 1 h.

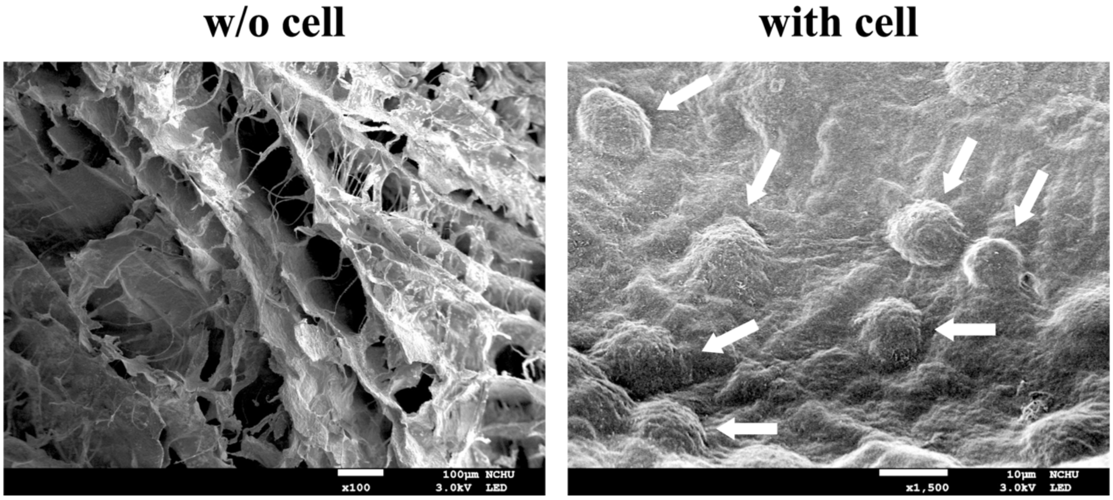

Figure S2. The microstructure of LdECM and cell-laden LdECM bioink after gelation for 1 day. White arrows point to HCC827 cells.

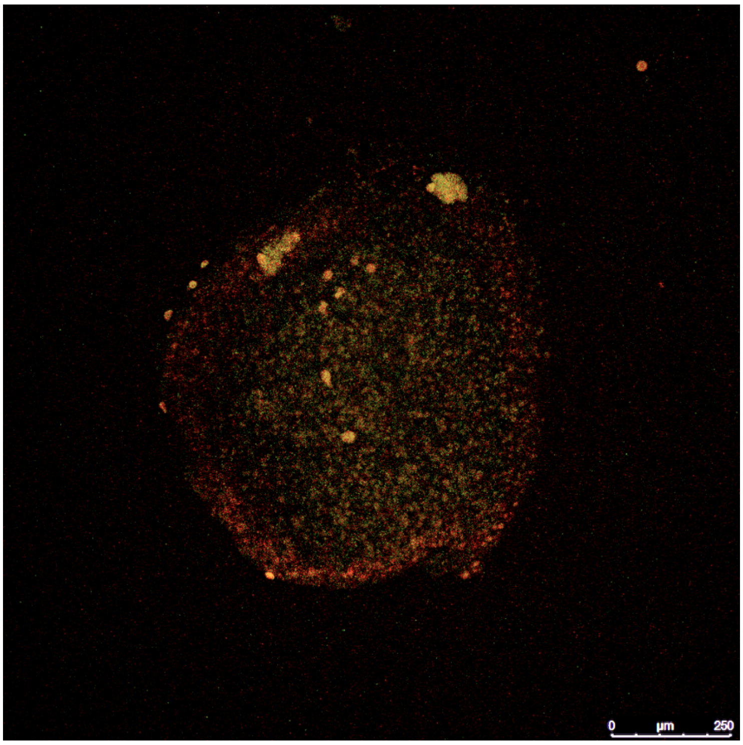

Figure S3. The pH differences of HCC827 cell spheroids from the center to the periphery were analyzed using fluorescent reagents. pH-dependent emission shifts from yellow-orange to deep red fluorescence under acidic and basic conditions.

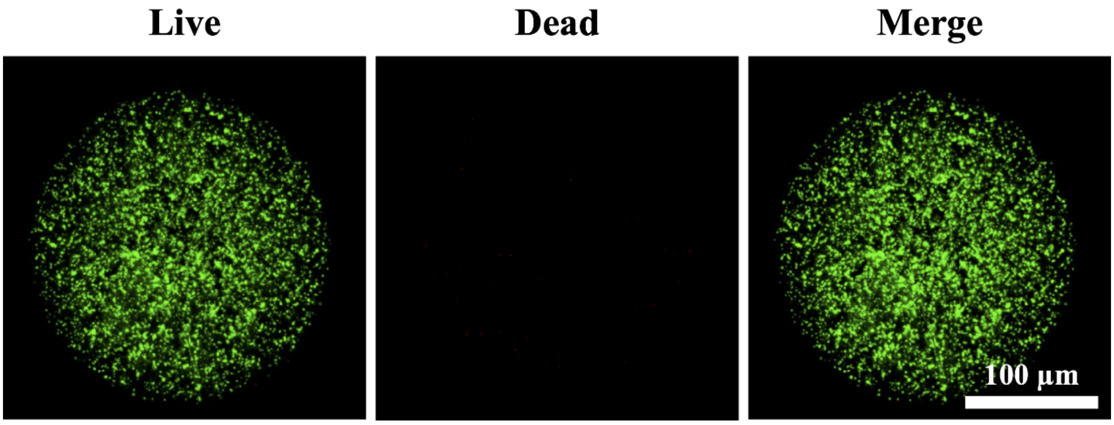

Figure S4. Live (green)/dead (red) staining of THP-1 cell-laden LdECM bioink after culture for 2 days. The scale bar is 100 µm.

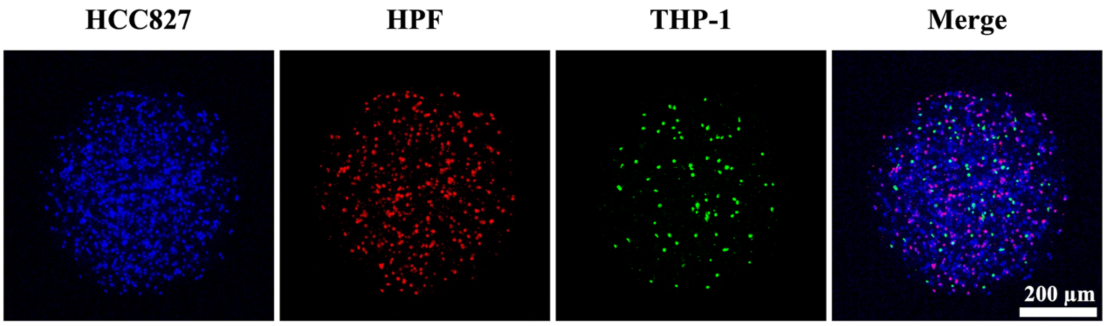

Figure S5. In order to simulate cancer as immune cells in the environment, we mixed HCC827 (blue), HPF (red), and THP-1 (green) in LdECM bioink and co-cultured for 3 days. The scale bar is 200 μm.

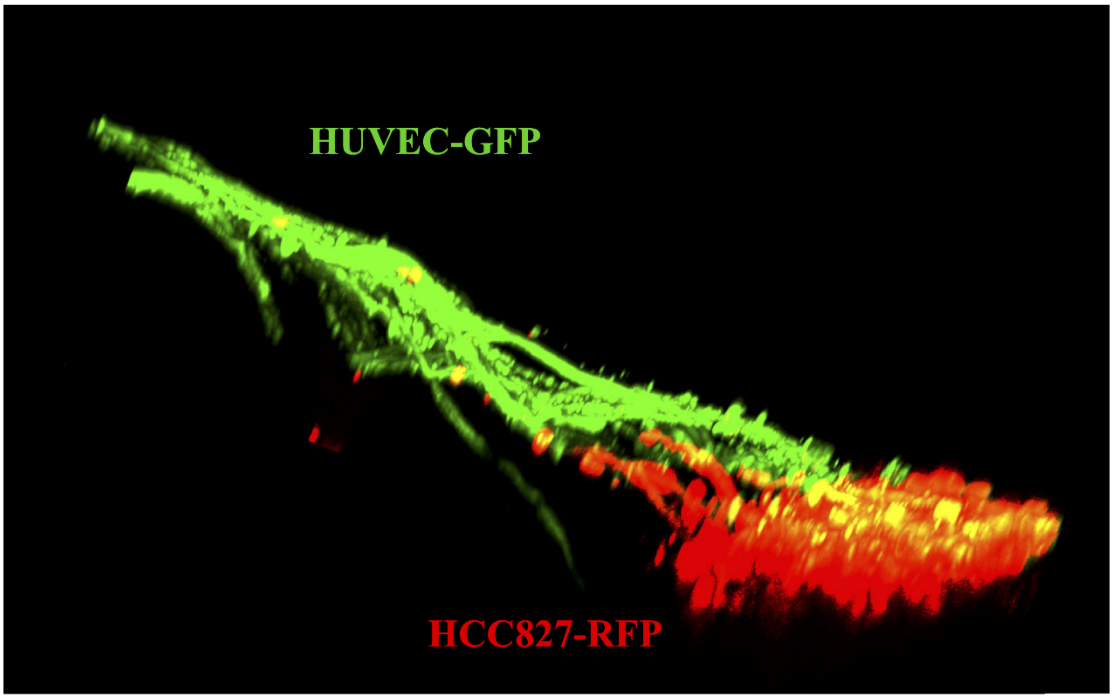

Figure S6. Endothelial cells result in the formation of a vascular barrier, which gravitates to the underlying cancer cell spheroids. A blood-vessel-like tissue grows in cancer cell spheroids.

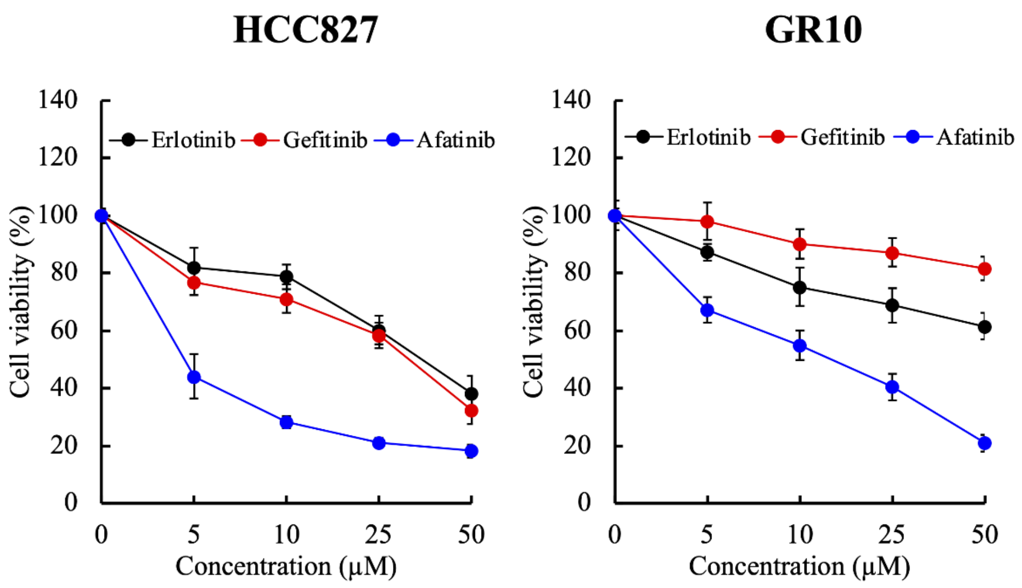

Figure S7. HCC827- and GR10-derived spheroids on a model are used to assess the toxicity after treated TKIs for 5 days. All experiments were performed in triplicate, and data are presented as mean ± SEM, n = 6 for each group.

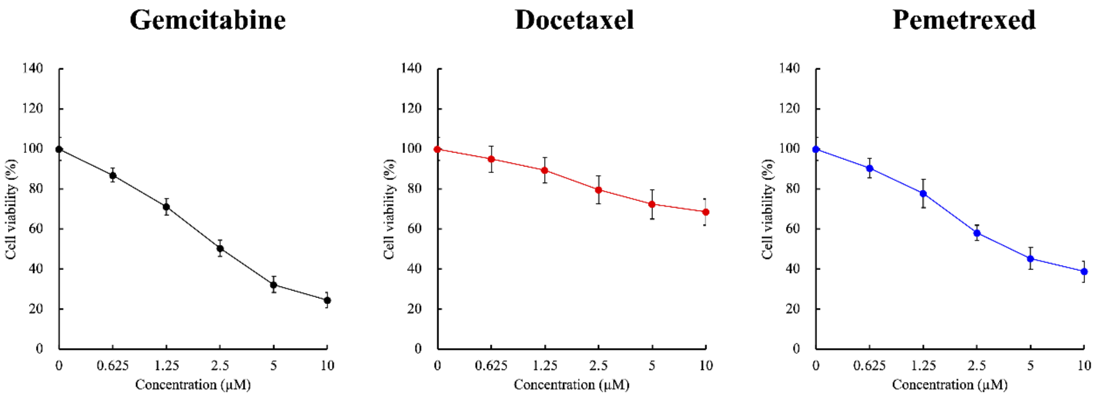

Figure S8. Various cell-derived spheroids on a model are used to assess the toxicity of HCC827 cell for various chemotherapy drugs. All experiments were performed in triplicate, and data are presented as mean ± SEM, n = 6 for each group.

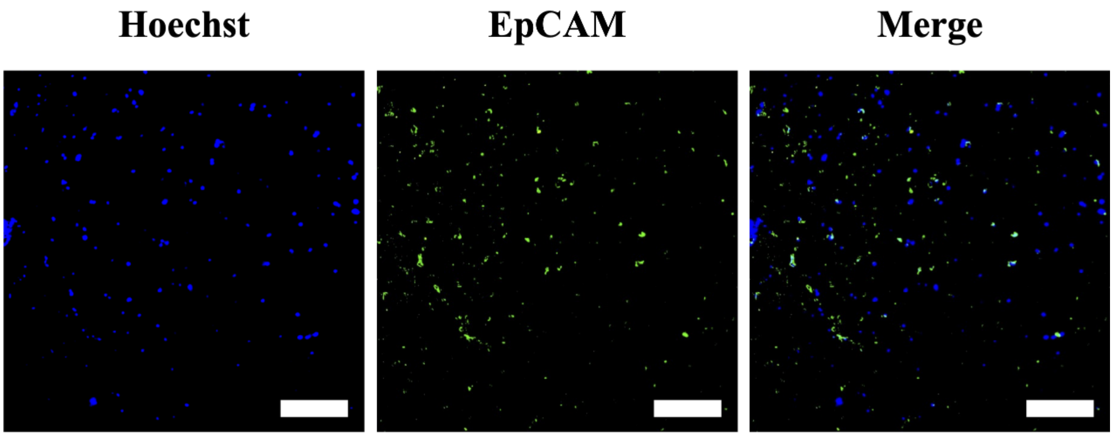

Figure S9. Cells isolated from the biopsy tissue are identified using immunofluorescence staining for specific lung cancer biomarkers (EpCAM, green). Approximately 76% of the cells reveal EpCAM and are presumed to be lung cancer cells. Those cells that do not show fluorescence may be cancer-associated fibroblasts or stromal cells (DAPI only)

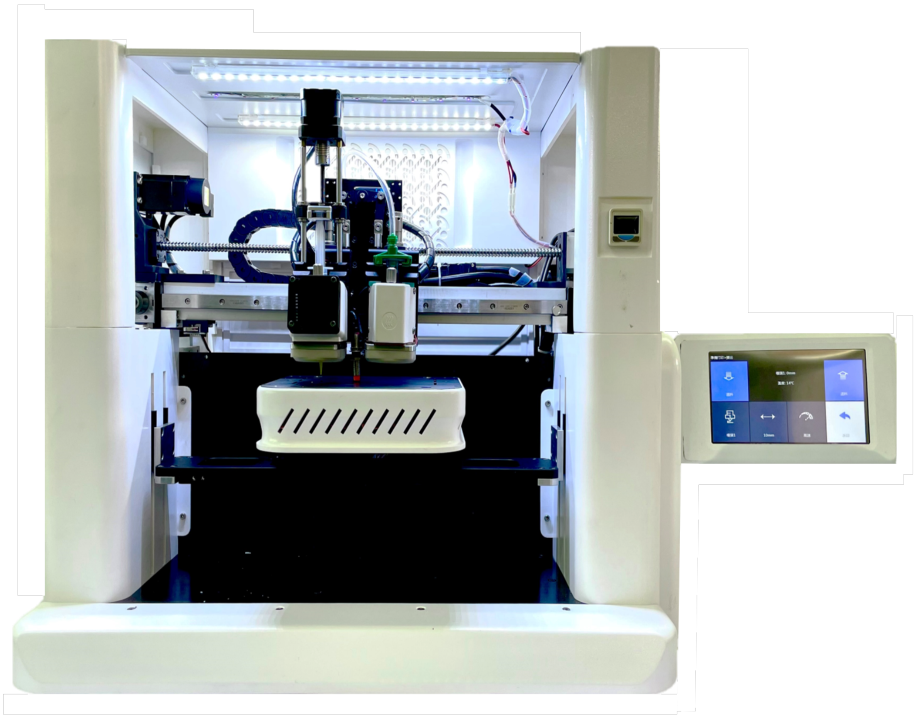

Figure S10. The tumor tissue spheroid bioprinter named "BioSmart Ver.1.0" that designed and manufactured by the laboratory.
